# Detecting Early Degradation of Wood Ultrastructure with Nonlinear Optical Imaging and Fluorescence Lifetime Analysis

**DOI:** 10.3390/polym16243590

**Published:** 2024-12-22

**Authors:** Alice Dal Fovo, Riccardo Cicchi, Claudia Gagliardi, Enrico Baria, Marco Fioravanti, Raffaella Fontana

**Affiliations:** 1 National Research Council—National Institute of Optics, Largo E. Fermi, 6, 50125 Florence, Italy; riccardo.cicchi@ino.cnr.it (R.C.); raffaella.fontana@ino.cnr.it (R.F.); 2 European Laboratory for Non-Linear Spectroscopy (LENS), Via Nello Carrara 1, 50019 Sesto Fiorentino, Italy; 3 Department of Agricultural, Food, Environmental and Forestry Sciences and Technologies (DAGRI), University of Florence, Piazzale delle Cascine, 18, 50144 Florence, Italy; claudia.gagliardi@unifi.it (C.G.); marco.fioravanti@unifi.it (M.F.); 4 Department of Physics and Astronomy, University of Florence, Via Sansone, 1, 50019 Sesto Fiorentino, Italy; enrico.baria@unifi.it

**Keywords:** two-photon excited fluorescence (TPEF), second harmonic generation (SHG), fluorescence lifetime imaging microscopy (FLIM), lignin, cellulose, hemicellulose, wood deterioration, delignification

## Abstract

Understanding the deterioration processes in wooden artefacts is essential for accurately assessing their conservation status and developing effective preservation strategies. Advanced imaging techniques are currently being explored to study the impact of chemical changes on the structural and mechanical properties of wood. Nonlinear optical modalities, including second harmonic generation (SHG) and two-photon excited fluorescence (TPEF), combined with fluorescence lifetime imaging microscopy (FLIM), offer a promising non-destructive diagnostic method for evaluating lignocellulose-based materials. In this study, we employed a nonlinear multimodal approach to examine the effects of artificially induced delignification on samples of Norway spruce (*Picea abies*) and European beech (*Fagus sylvatica*) subjected to increasing treatment durations. The integration of SHG/TPEF imaging and multi-component fluorescence lifetime analysis enabled the detection of localized variations in nonlinear signals and τ-phase of key biopolymers within wood cell walls. This methodology provides a powerful tool for early detection of wood deterioration, facilitating proactive conservation efforts of wooden artefacts.

## 1. Introduction

Wood is one of the most widely used materials for art production due to its versatility and availability. A comprehensive understanding of wood alteration processes and the early identification of decay effects are essential for developing conservation strategies that can extend the lifespan of wooden artworks. Characterizing deterioration-induced changes at the ultrastructural and molecular levels can, on the one hand, help halt or even reverse these processes before they compromise the macroscopic physical properties of the objects; on the other hand, it enables the understanding of techniques employed by craftsmen to deliberately alter material properties, such as the artificial aging treatments used by luthiers to enhance the acoustic properties of musical instruments by modifying the dynamic viscoelastic properties of the wood [1,2].

Wood deterioration can result from various factors, some of which are not fully understood. The degradation may affect the cell walls at ultrastructural level, where three main biopolymers are arranged in a complex matrix, with their relative proportions varying depending on the wood species: cellulose (40–44%), hemicellulose (15–32%), and lignin (18–35%). Other components, including proteins, fatty acids, pectins, starch, and various polysaccharides and sugars, are also present, typically comprising 2–15% of the wood mass [3,4]. Cellulose is arranged in discrete units known as elemental fibrils, with the inner core comprising macromolecules tightly packed into crystalline structures and the outer layers consisting of amorphous regions. Lignin is a heteropolymer of repeated phenylpropane units with a diversity of bonds among three variants of the monomeric form that makes lignin extremely resistant to degradation. Hemicellulose is a branched heteropolymer of pentose and hexose sugar monomers characterized by more amorphous regions than cellulose. Hemicellulose acts as the link between cellulose and lignin and its degradation profoundly affects the ability of lignocellulose to behave as a rigid structural system [5].

The main mechanisms involved in biopolymer degradation, such as depolymerization, oxidation and hydrolysis reactions, may impact each molecule differently. Regarding biological degradation, various microorganisms—including fungi, and bacteria—utilize enzymatic systems to break down the lignin, thereby accessing the cellulose [6]. Polysaccharides in biomass serve as an excellent energy source not only for microorganisms: recent research in renewable energy has been focused on the biochemical conversion of wood polysaccharides into biofuels, providing a sustainable alternative to fossil fuels [7]. Several methods have been proposed to degrade lignin and improve the accessibility of biomass to enzymes for the saccharification of cellulose and its subsequent fermentation into ethanol [8]. One of the pretreatment methods consists of wood incubation in a solution of hydrogen peroxide and acetic acid (HPAC). In this process, hydrogen peroxide and acetic acid react and form peracetic acid, which can convert the aromatic ring of lignin into carboxylic acid and their lactones by oxidative cleavage [9]. HPAC stands out with its high delignification ability without the use of high temperatures or strong acids [10,11,12] and has been used for a long time in the pulp and paper industry as a plant fiber isolating process [13].

Advanced imaging techniques are currently being explored to study the effect of chemical changes on the structural and mechanical properties of wood [14]. Nonlinear optical microscopies (NLOM), such as two-photon excited fluorescence (TPEF) and second harmonic generation (SHG), enable label-free and non-destructive analysis of morphology and chemical changes in the ultrastructure of cells, with tomographic capabilities and better penetration depth than linear optical microscopies. Recent studies suggest that the effects of wood degradation can be monitored based on variations in TPEF and SHG signals, as cellulose, hemicellulose, and lignin exhibit nonlinear properties [15]. Cellulose has the potential to generate an SHG signal due to the non-centrosymmetric nature of its constituent molecules. The hierarchical structure and parallel alignment of cellulose chains within the crystalline regions make microfibrils highly effective generators of second harmonic signals [16]. Therefore, the intensity of the SH signal may vary due to changes in the arrangement of molecular chains during aging, such as changes in cellulose crystallinity and the size of the cellulose crystallites and/or tighter packing between cellulose and glucomannans [17]. Lignin shows a characteristically strong autofluorescence in the visible spectral region due to the presence of multiple fluorophore types [18,19], which can be excited through nonlinear absorption using a NIR excitation wavelength (beyond 800 nm), with a broad emission between 400 and 700 nm [20].

Fluorescence is highly sensitive to the molecular environment, including pH changes, thermal exposure, and chemical treatments, making it an effective indicator for monitoring decay-induced alterations in wood cells [21]. While the autofluorescence of lignin has been well studied, the autofluorescence properties of cellulose remain largely unexplored. However, recent findings have yielded promising results that warrant further investigation and detailed characterization [22]. Olmstead and Gray [23] report that cellulose exhibits a relatively strong characteristic emission and that the removal of lignin from pulp samples via acidic chlorite treatment does not significantly reduce fluorescence. One possible explanation for the absorbance of cellulose is that the molecule chains interact with each other in the solid state, forming absorbing centers [24]. Kapsokalyvas et al. recently demonstrated that in addition to lignin and cellulose, hemicelluloses extracted and isolated from various plant cell walls exhibit autofluorescence [25]. Autofluorescence was excited nonlinearly at 820 nm, resulting in emission peaks centered at 510 nm, 520 nm, and 580 nm for cellulose, hemicellulose, and lignin, respectively. This indicates that biomass fluorescence is influenced by its local composition and represents a composite signal from its components. Furthermore, the fluorescence intensity of individual polymers can be influenced by their interactions with other polymers [26]. For instance, covalent bonding between lignin and hemicellulose has been shown to suppress lignin fluorescence, whereas the removal of carbonyl groups tends to enhance it [14,27]. As a result, the extraction of lignin from wood inevitably modifies its autofluorescence properties. By analyzing the emission decay dynamics of fluorophores in wood biomass, the specificity of autofluorescence measurements can be improved, providing a valuable tool for early-stage degradation detection [25]. According to Kapsokalyvas et al., the average fluorescence lifetimes for unmodified lignin, hemicellulose, and cellulose are 0.24 ± 0.01 ns, 2.01 ± 0.25 ns, and 2.16 ± 0.06 ns, respectively.

In this study, we analyzed two wood species, Norway spruce (*Picea abies*) and European beech (*Fagus sylvatica*), representing softwood and hardwood, respectively. These species were chosen for their widespread use in artefact production, owing to their excellent physical properties and broad availability across Europe. The samples underwent delignification treatment using HPAC with varying incubation periods. This chemical treatment mimics some of the effects of fungal attack, making it suitable for investigating biological wood decay. The progressive removal of lignin, the primary fluorescence emitter in wood biomass, enables the assessment of the autofluorescence properties of other biopolymers, particularly hemicelluloses, whose depolymerization is often an early indicator of aging phenomena. Fluorescence spectroscopy was employed to track changes in fluorescence emission at increasing treatment exposure. Furthermore, non-linear optical imaging, combining TPEF-SHG and FLIM analysis, was conducted to explore the potential for detecting early-stage degradation.

## 2. Materials and Methods

### 2.1. Wood Samples and Chemical Treatment

Transverse sections (block dimension approximately 5 × 5 × 5 mm^3^) of spruce (*Picea abies*) and beech (*Fagus sylvatica*) wood were taken from the outer part of logs (mature wood) and sliced in cross sections to a thickness of approximately 20 µm. Each sample was subjected to a delignification (DL) treatment by immersion in a 1:1 solution of hydrogen peroxide and acetic acid (HPAC) with increasing incubation time: 0 h (control sample), 4 h, 8 h, 10 h, 12 h, 16 h, 24 h, 36 h, 48 h, 120 h. The slices were then rinsed with demineralized water and sandwiched between a microscope slide and a coverslip for the analysis.

### 2.2. Fluorescence Spectroscopy Setup

The setup [28] allows for fluorescence, reflectance and Raman spectroscopies. It consists of a spectrometer, a custom fiber bundle probe (EMVision LCC, Loxahatchee, FL, USA) and four light sources. Fluorescence spectroscopy can be performed using two different laser diodes as excitation sources (TEC 042, Sacher Lasertechnik, Maburg, Germany) emitting at 378 nm and at 445 nm. The light sources are coupled to optical fibers for light delivering and collection, with core diameters of 100 μm and 300 μm, respectively. The detection system is based on a monochromator (MicroHR, HORIBA Scientific, Edison, NJ, USA) equipped with a 600 lines/mm grating. Emission filters are switched using a motorized wheel according to the excitation source: a long-pass filter at 400 nm (Longpass 400 nm, Melles Griot, Albuquerque, NM, USA) for fluorescence excited at 378 nm; and a long-pass filter at 458 nm (LP02-458RS-25, Semrock, Rochester, NY, USA) for fluorescence excited at 445 nm. In this analysis, each fluorescence spectrum is the average of three spectra acquired on the sample’s surface in different positions.

### 2.3. Nonlinear Optical Microscope

The microscope was developed by CNR-INO based on a dual output fs-laser (Chameleon Discovery, Coherent Inc., Saxonburg, PA, USA) that allows for multiple NLO contrast mechanisms, including TPEF, SHG, coherent anti-Stokes Raman scattering (CARS), and FLIM. In this application, the excitation wavelength was set at 840 nm, with a pulse duration of 100 fs and a repetition rate of 80 MHz. The laser beam was raster scanned using two galvanometric mirrors (Cambridge Technology, Bedford, MA, USA) and focused onto the sample using a 20× water immersion objective lens (NA 0.95, WD 0.25 mm, Carl Zeiss Microscopy, Jena, Germany). The average power was measured to be approximately 15 mW immediately after the objective lens. The SHG signal was collected in a forward configuration and selectively detected by a passband filter centered at 420 ± 10 nm (Semrock FF01-420/10-25). The autofluorescence signal was epi-collected and filtered at 505 ± 119 nm (FF01-505/119-25, Semrock, Rochester, NY, USA). Both signals were directed to two photomultiplier tubes (PMT, H7422-40, Hamamatsu, Hamamatsu, Japan) for parallel and co-registered SHG and TPF imaging. Each sample was measured over three different areas (size = 300 × 300 μm^2^, image resolution = 512 × 512 pixels). Axial stacks (20 images) were also acquired over a 50 µm axial range. The laser power and gain settings for detecting TPEF and SHG signals were standardized across all measurements.

FLIM images were acquired in the same areas analyzed with SHG and TPF imaging. Two-photon excited autofluorescence was revealed using a photomultiplier tube (PMH-100, Becker & Hickl GmbH, Berlin, Germany) routed to a time-correlated single-photon counting (TCSPC, SPC-150, Becker & Hickl) acquisition card that records the fluorescence intensity decay for each pixel. The integration time for each FLIM image was 80 s. The SH signal was excluded from the acquisition using a dichroic mirror. The fluorescence lifetime was analyzed using SPCImage NG software v.8.9 (Becker & Hickl), which employs an iterative convolution process based on a maximum-likelihood estimation (MLE) algorithm to calculate the decay function parameters for each pixel in the FLIM image. The MLE approach maximizes the probability that the model function accurately represents the decay data. Compared to the commonly used weighted least-squares method, MLE offers improved fitting accuracy, particularly in conditions with low photon counts, and avoids the bias toward shorter lifetimes that is inherent in least-squares fitting.

## 3. Results and Discussion

**Figure 1:**
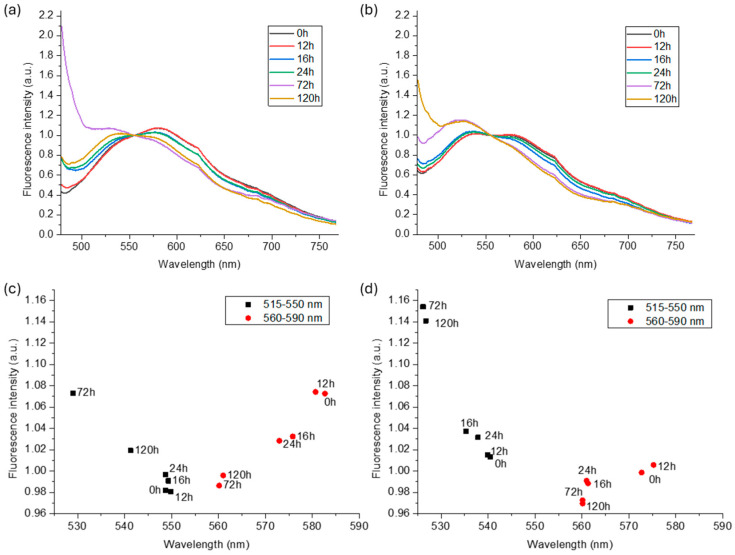
Normalized averaged fluorescence spectra (λ_ext_ = 445 nm) of spruce (**a**) and beech (**b**) and scatter plots of the peaks of the two main emission bands for spruce (**c**) and beech (**d**) at increasing treatment time.

Fluorescence spectra were obtained from both wood species subjected to delignification (DL) at increasing treatment durations. Given the wavelength of the femtosecond laser used for NLO microscopy (840 nm), it was expected that two-photon absorption would produce fluorescence spectra comparable to those measured with one-photon absorption at 445 nm. To accurately compare the relative intensities of the different fluorophores and minimize potential bias or interference from background signals, normalization was performed in the spectral region between the two main emission peaks, specifically at 555 nm [29]. The normalized averaged spectra for both spruce and beech (Figure 1a,b, respectively) display two prominent peaks: one in the range of 515–550 nm and another between 560 and 590 nm, attributed to hemicellulose–cellulose and lignin, respectively, in agreement with the literature [25]. With increasing DL, both species showed an increase in the relative intensity of hemicellulose–cellulose signals and a decrease in lignin-related fluorescence. This suggests that the treatment-induced lignin reduction not only suppresses lignin fluorescence but also increases the relative contribution of the other two components. Moreover, the peak positions of the main emission bands (515–550 nm and 560–590 nm) shift toward shorter wavelengths as the treatment time increases (see Figure 1c,d for spruce and beech, respectively). The blue shift in the maxima of both emission bands may be associated with the rearrangement of the molecular interaction and pH changes in the molecular environment: lignin and cellulose have both been reported to exhibit pH-dependent emission [30]. It is also possible that the tail of the hemicellulose–cellulose emission band, which partially overlaps with the lignin emission band, contributes increasingly to the blue shift as lignin is leached.

NLO imaging allowed for the visualization of the distribution of autofluorescence and SHG signal over the wood ultrastructure. All samples were measured with well-defined laser power and detection settings to obtain comparable results. TPEF and SHG signals were detected and displayed as intensity greyscale images, which were then merged using Java-based image processing software (ImageJ Version 1.54). To simultaneously display their spatial distribution, color-composite images were generated assigning the red and green color channels to TPEF and SHG signals, respectively. Both spruce and beech samples showed a noticeable increase in SHG signal intensity with prolonged treatment exposure, becoming particularly evident after 48 h.

**Figure 2:**
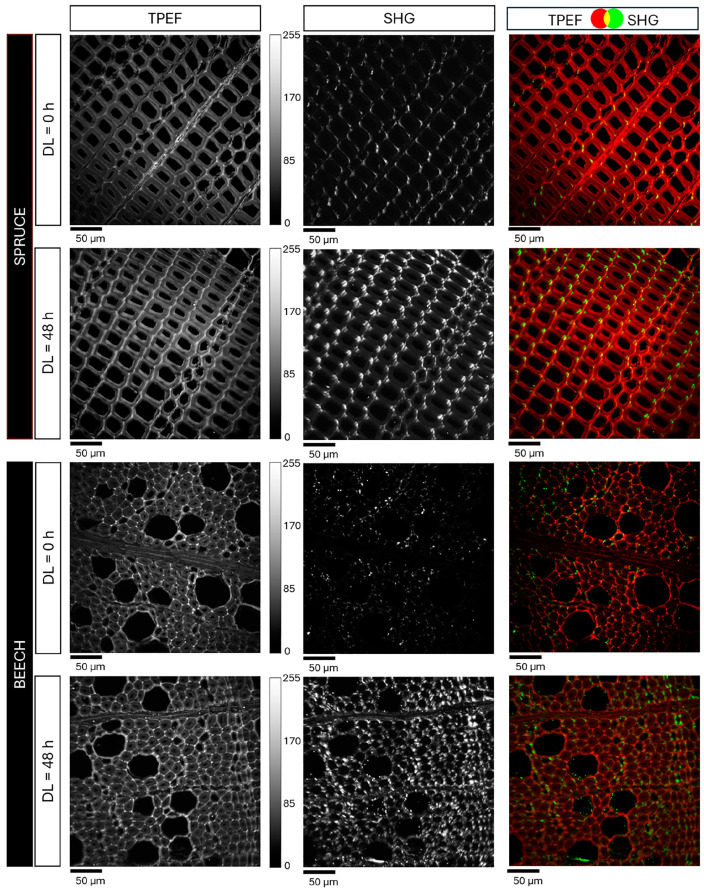
TPEF and SHG intensity images (300 × 300 µm^2^, 512 × 512 pixel) of spruce and beech samples, acquired before (DL = 0 h) and after the delignification treatment (DL = 48 h). The first and second columns show eight-bit grayscale images, representing the SHG and TPEF signal intensities as pixel brightness within the dynamic range of 0–255. The third column presents color-composite images where the SHG signal, primarily associated with cellulose, and the TPEF signal are displayed in green and red, respectively.

Figure 2 shows eight-bit greyscale images of spruce and beech samples acquired at 0 h and 48 h of treatment, together with the merged SHG/TPEF images. Signal intensities are represented as pixel brightness within a dynamic range of 0–255. While no significant changes in fluorescence intensity are observed, the second harmonic signal, primarily associated with cellulose, is significantly more intense and widespread in the samples treated for 48 h compared to untreated ones. This could be related to a reduction in the self-absorption of the SH signal by lignin, which is leached during the treatment. On the other hand, a decrease in the fluorescence intensity would have been expected following delignification. However, given the 445–565 nm detection range, the reduction in lignin contribution, although substantial in the biomass, may be counterbalanced by the fluorescence of other components such as cellulose and hemicellulose, which remain unaffected by the delignification reaction. These components may also experience less quenching as lignin is progressively removed from the cell wall biomass.

**Figure 3:**
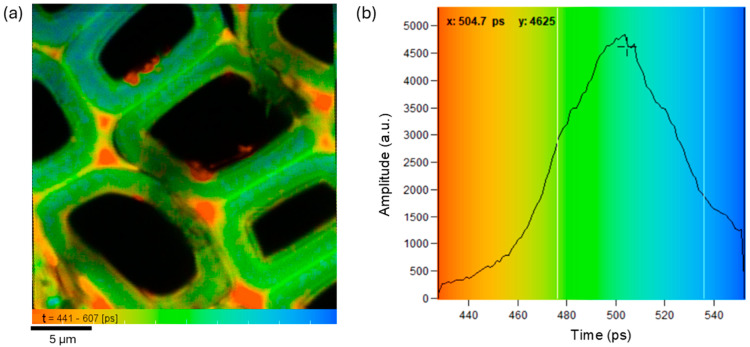
FLIM analysis of the untreated spruce sample: color-coded lifetime image (25 × 25 µm^2^) with the mean lifetime values $\tau_{1-3}$ of the decay components (**a**), resulting from the triple-exponential fit of the experimental data; amplitude-weighted lifetime (**b**) showing how often the pixels with specific lifetime values occur in the matrix decay image (**a**).

Time-resolved fluorescence analysis was initially carried out on the untreated spruce sample to identify the three primary decay components, which could be attributed to lignin, hemicellulose, and cellulose. Three regions (25 × 25 µm^2^) were examined using an objective lens with NA = 1.4 to highlight differences in the cell walls. The results obtained on one area are presented in Figure 3. The fluorescence decay signal was modeled using a triple-exponential function (Equation (1)), optimized through maximum likelihood estimation, reduced chi-square,$\;\chi_{r}^{2}$ = 1.09:

(1) $$f\left(t\right)=a_{1}e^{-t/\tau_{1\;}}+a_{2}e^{-t/\tau_{2}}+a_{3}e^{-t/\tau_{3}},$$

where $\tau_{1-3}$ and $a_{1-3}$ are the lifetimes and the amplitudes of the three predominant decay components, respectively. The amplitude-weighted lifetime from the triple-exponential fit (Figure 3b) represents the distribution of pixel lifetimes in the color-coded fluorescence lifetime image (decay matrix) shown in Figure 3a, where the overall mean lifetime is $\tau_{m}$ = 514 ps (σ = 25 ps). From the FLIM image, it is possible to spatially resolve three main lifetimes, as shown in blue, green and orange colors. The three lifetimes occur in the middle lamella and the secondary cell wall. Lifetimes in the middle lamella are shorter (orange) than those in the secondary walls (green and blue). The mean lifetime values for the decay components resulting from the fit are indicated in regions of the FLIM image corresponding to pixels with the maximum amplitude-weighted lifetime (highlighted by a cross in the decay matrix). These values are approximately $\tau_{1}$ ≈ 180 ps, $\tau_{2}$ ≈ 840 ps, and $\tau_{3}$ ≈ 2230 ps, with respective amplitudes of $a_{1}$ ≈ 79.1%, $a_{2}$ ≈ 15.6 %, and $a_{3}$ ≈ 5.3%. These values are consistent with those reported in the literature for lignin, hemicellulose, and cellulose, respectively [23].

**Figure 4:**
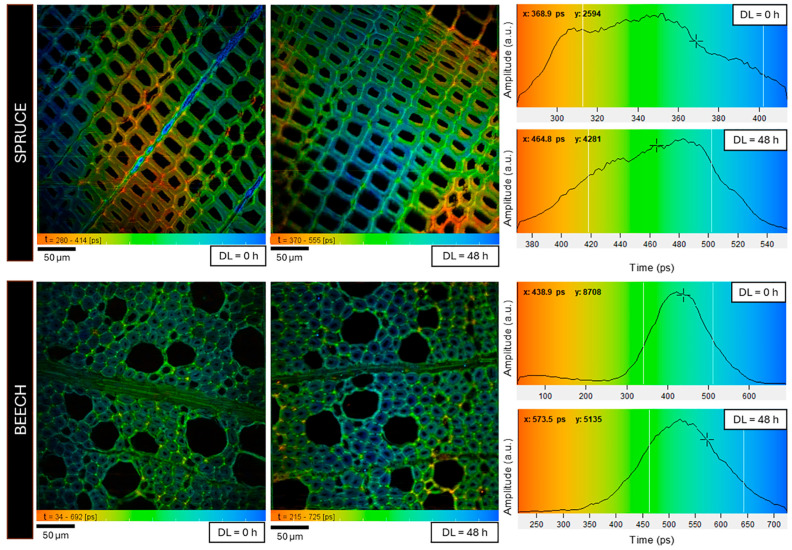
FLIM results on spruce and beech samples, acquired before (DL = 0 h) and after the delignification treatment (DL = 48 h): color-coded lifetime images (300 × 300 mm^2^, 512 × 512 pixel) and amplitude-weighted lifetime distributions.

Once we determined the lifetime and amplitude values of the three main fluorescent contributors in spruce cell walls, we used the same procedure to systematically analyze the delignified samples of both wood species to verify possible changes in $\tau_{1-3}$ and $a_{1-3}$ as the treatment exposure increases. For all samples, FLIM measurements were performed on three regions of each sample (300 × 300 μm^2^), i.e., the same areas previously examined with SHG-TPEF. Lifetime images and amplitude-weighted lifetime distribution of spruce and beech samples at DL = 0 h and DL = 48 h are reported in Figure 4 as an example.

**Figure 5:**
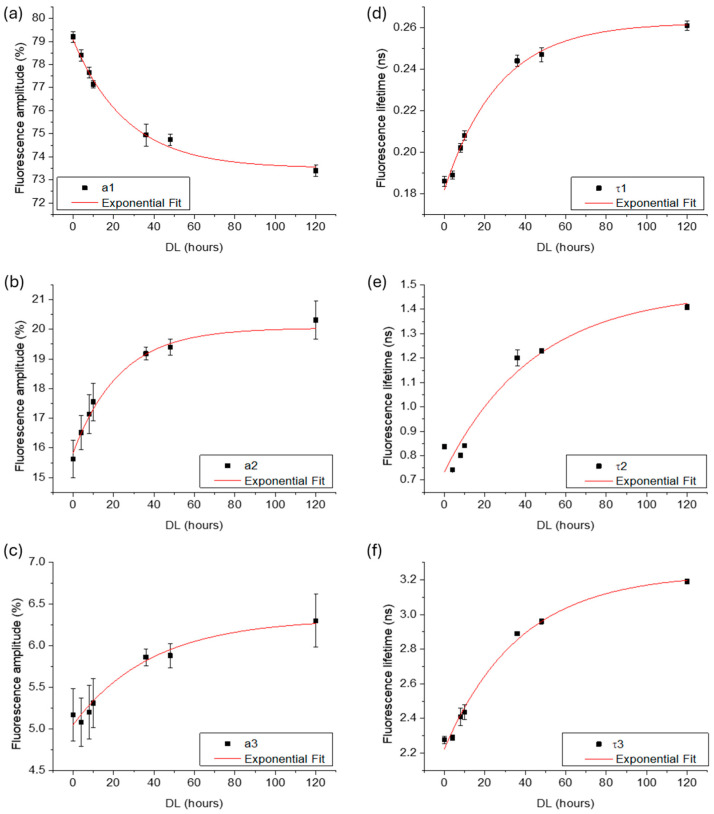
Scatter plots of average amplitudes (**a**–**c**) and lifetimes (**d**–**f**) for the three primary decay components, attributed to lignin, hemicellulose and cellulose, respectively, measured in spruce samples exposed to increasing hours of delignification. Error bars represent the standard deviation, and red lines indicate the exponential fit of the experimental data.

**Figure 6:**
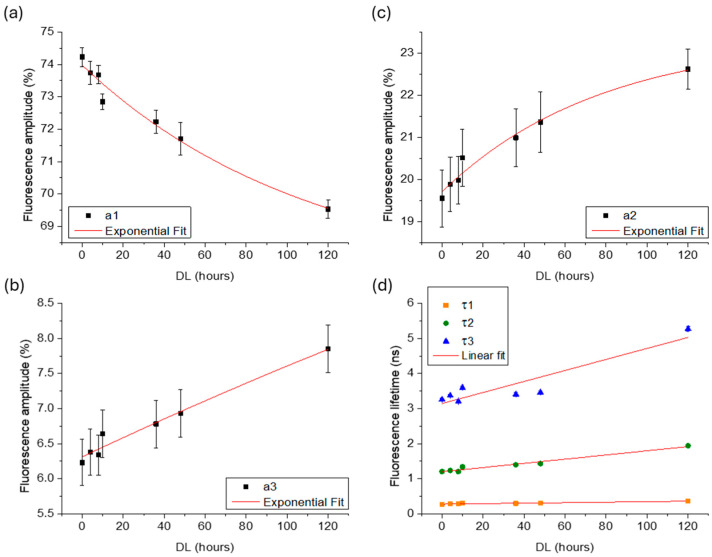
Scatter plots of average amplitudes (**a**–**c**) and lifetimes (**d**) for the three primary decay components, attributed to lignin, hemicellulose and cellulose, respectively, measured in beech samples exposed to increasing hours of delignification. Error bars represent the standard deviation, and the red lines indicate the exponential ($a_{1-3}$) and linear ($\tau_{1-3}$) fit of the experimental data.

The average fluorescence lifetime and amplitude values were calculated and plotted as a function of delignification time (0, 4, 8, 10, 36, 48, and 120 h). Figure 5 and Figure 6 show the results for spruce and beech samples, respectively. As expected, the delignification treatment led to a decrease in the fluorescence amplitude of lignin, along with a corresponding increase in the amplitudes for hemicellulose and cellulose. For both species, an exponential function provided the best fit for the experimental data, as defined by Equation (2):

(2) $$y=y_{0}+Ae^{-\left(x-x_{0}\right)/t},$$

where y represents the fluorescence amplitude ($a_{1-3}$), $y_{0}$ is the offset, *A* is the initial value, $x_{0}$ is initial time, x is the delignification time, and t is the decay constant. For spruce, the decay constant t was 28 h, 23 h, and 48 h for lignin, hemicellulose, and cellulose, respectively, while for beech, the t values were 112 h, 77 h, and 719 h, respectively.

In terms of fluorescence lifetime, both species exhibited an increase in the τ values of the three fluorophores with increasing DL time. For spruce, the *τ* values followed an exponential growth, resulting in t values of 27 h, 44 h, and 37 h for lignin, hemicellulose, and cellulose, respectively. In contrast, for beech, the lifetime values were best described by a linear function, with slopes of 0.016, 0.006, and 0.001 for lignin, hemicellulose, and cellulose, respectively.

## 4. Conclusions

The multi-analytical method used in this study, combining linear fluorescence spectroscopy, nonlinear optical imaging, and fluorescence lifetime, enabled the identification of preliminary indicators of the changes that occur at the ultrastructural level in two wood species during the delignification process.

Two primary emission bands contributing to the fluorescence signal were identified within spectral ranges compatible with those of hemicellulose/cellulose and lignin reported in the literature [25]. A blue shift in the peak positions of the main emission bands was also observed as the treatment time increased. A concurrent increase in the intensity of the SHG signal of cellulose was detected, which can be explained by the diminishing reabsorption effects from lignin, progressively leached out by the treatment. Simultaneously, no significant differences were noted in the intensity of two-photon excited fluorescence (TPEF) as the delignification time increased. This may be attributed to an enhanced contribution from other fluorophores, whose fluorescence undergoes less quenching as lignin is progressively removed from the cell wall biomass. The three main components of the fluorescence decay signal were also identified, which, in agreement with previous studies, can be attributed to the three predominant biopolymers in wood biomass, namely lignin, hemicellulose and cellulose. Three main contributions in the fluorescence lifetime were identified through tri-exponential analysis of fluorescence decay, yielding lifetime (*τ*) and amplitude (*a*) parameters for each of the three decay components, which were associated with lignin, hemicellulose, and cellulose, as supported by the literature. This analysis enabled the characterization of the relative variations in the fluorescence components as the treatment duration increased. Although fluorescence lifetime is independent of fluorescence intensity and fluorophore concentration [31], it is sensitive to the structure and composition of the fluorophores, as well as to the presence of interacting surrounding molecules. Consequently, modifications in fluorescence lifetime indicate changes in the chemical composition and organization of the primary fluorescent polymer, lignin, and alterations in the interactions with other polymers, particularly hemicellulose [32].

The observed variations in fluorescence emission, intensity of nonlinear optical signals and fluorescence lifetime parameters with increasing hours of treatment suggest that this approach can be effectively used in art conservation to detect the effects of decay on wooden artefacts at an early stage after calibration on naturally aged material. To further elucidate the mechanisms underlying the observed changes, vibrational and nuclear spectroscopy methods can offer detailed insights into the molecules involved. We intend to broaden our analysis by including a larger number of samples with varying delignification durations, thereby enhancing the statistical significance of the extracted data.

## Data Availability

The original contributions presented in this study are included in the article. Further inquiries can be directed to the corresponding author.
